# Temporal Trends in Patients with Peripheral Artery Disease Influenced by COVID-19 Pandemic

**DOI:** 10.3390/jcm11216433

**Published:** 2022-10-30

**Authors:** Karsten Keller, Volker H. Schmitt, Lukas Martin Alois Hobohm, Christoph Brochhausen, Thomas Münzel, Christine Espinola-Klein

**Affiliations:** 1 Department of Cardiology, University Medical Center Mainz, Johannes Gutenberg-University Mainz, 55131 Mainz, Germany; 2 Center for Thrombosis and Hemostasis (CTH), University Medical Center Mainz, Johannes Gutenberg-University Mainz, 55131 Mainz, Germany; 3 Department of Sports Medicine, Medical Clinic VII, University Hospital Heidelberg, 69120 Heidelberg, Germany; 4 German Center for Cardiovascular Research (DZHK), Partner Site Rhine Main, 55131 Mainz, Germany; 5 Institute of Pathology, University of Regensburg, 93053 Regensburg, Germany

**Keywords:** COVID-19, peripheral artery disease, time-trends, amputation, intervention

## Abstract

**Background:** The COVID-19 pandemic influences the health care management of acute and chronic diseases. Data concerning the influence of the pandemic on hospitalizations of patients with peripheral artery disease (PAD) are sparse. **Methods:** We analysed all patients hospitalized due to PAD between 1 January 2019 and 31 December 2020 in Germany. Hospitalizations of PAD patients during the pre-pandemic year 2019 were compared to the pandemic year 2020. **Results:** Overall, 361,029 hospitalizations of PAD patients in the years 2019 and 2020 (55.4% aged ≥70 years; 36.6% females) were included in this study. In the pre-pandemic year of 2019, a total of 25,501 (13.2%) more hospitalizations due to PAD were detected compared to the COVID-19 pandemic year of 2020 (2019: 192,765 [53.4%] vs. 2020: 168,264 [46.6%], *p* = 0.065). Overall, in 610 (0.4%) of the hospitalization cases, a COVID-19 infection was diagnosed. Regarding interventional/surgical treatments, total numbers of peripheral endovascular intervention of the lower extremity decreased by 9.9% (83,845 vs. 75,519, *p* < 0.001), surgical peripheral artery revascularization of the lower extremity by 11.4% (32,447 vs. 28,754, *p* = 0.041) and amputations by 4.0% (20,612 vs. 19,784, *p* < 0.001) in 2020 compared to 2019. The case fatality rate (2.6% vs. 2.4%, *p* < 0.001), as well as MACCE rate (3.4% vs. 3.2%, *p* < 0.001), were slightly higher during the pandemic year 2020 compared to the pre-pandemic year 2019. **Conclusions:** The COVID-19 pandemic influenced the number of hospitalizations of PAD patients with a 13.2% reduction in hospital admissions and decreased total numbers of revascularization and amputation treatments.

## 1. Introduction

During December 2019, pneumonia cases caused by a previously unknown virus occurred in China [1,2,3,4]. In Germany, the first patient with SARS-CoV-2 infection was identified at the end of January 2020 in Bavaria [3,5]. This initial cluster of coronavirus disease 2019 (COVID-19) was the starting point of a sustained transmission of SARS-CoV-2 resulting in spread of COVID-19 across the German population [3,4,6].

The ongoing pandemic caused by SARS-CoV-2 has been associated with millions of deaths worldwide [3,7]. In order to encounter the increasing numbers of infected individuals the government of Germany established lockdown measures [3]. Studies have shown that admissions for acute cardiovascular events as well chronic cardiovascular diseases [8,9,10], but also for cancer [11,12,13] as well as mental/behaviour disorders and others [13] decreased during the pandemic. It was suggested that this reduction regarding the number of hospital admissions during the pandemic was probably multifactorial including patients’ fear of SARS-CoV-2 contagion and infection during hospitalization as one key factor [8,14]. Data regarding the impact of the COVID-19 pandemic on hospital admissions of patients with peripheral artery disease (PAD) are sparse [14].

A few studies revealed that admitted PAD patients during the COVID-19 pandemic showed increased severity of PAD and more acute complications [14,15]. This little and limited attention regarding the impact of the COVID-19 pandemic on the admission numbers of PAD patients is contrary to the large burden of PAD and the influence of PAD on morbidity and mortality worldwide [16,17].

## 2. Material and Methods

We selected and analysed all hospitalizations of patients with a main diagnosis of PAD (ICD-code I70.2) in Germany during the observational period between the 1 January 2019 and the 31 December 2020 (source: RDC of the Federal Statistical Office and the Statistical Offices of the federal states, DRG Statistics 2019–2020, and own calculations). Patients’ main diagnosis is defined as the diagnosis, which is mainly responsible for the hospitalization of the patient documented by the discharging physician [18,19].

In Germany, patients’ diagnoses are coded according to the established coding-guidelines ICD-10-GM (International Classification of Diseases, 10th Revision with German Modification) and diagnostic, surgical as well as interventional procedures are coded by established OPS codes (surgery, diagnostic and procedures codes: Operationen- und Prozedurenschlüssel) [19,20,21]. The Federal Statistical Office of Germany (Statistisches Bundesamt, Wiesbaden, Germany) gleans all data from hospitalized patients in Germany, which were coded and processed according to the diagnosis related groups [DRG] system [19].

The identified and included hospitalization–cases with PAD diagnosis were stratified for the treatment year in the pre-pandemic year 2019 and in the COVID-19 pandemic year 2020 and these years were compared.

### 2.1. Study Endpoints and in-Hospital Adverse Events

The primary study outcomes were defined as (reduced) number of hospitalizations of PAD patients, (reduced) numbers of revascularization treatments and amputations. The secondary study outcome comprised in-hospital case fatality rate and major adverse cardiac and cerebrovascular events (MACCE, composite outcome of all-cause in-hospital death, acute myocardial infarction [MI, ICD-code I21], and/or ischemic stroke [ICD-code I63]).

#### Definitions

According to the recommendations of the WHO (World Health Organization), obesity was defined as a body mass index ≥30 kg/m^2^ [22]. Shock as well as cardio-pulmonary resuscitation were defined according to current European guidelines [23,24,25]. In this study, major amputations comprised surgeries with amputations above the ankle (OPS-code: 5-864) and minor amputations were defined as surgeries comprising amputations below the ankle (OPS-code: 5-865). Amputations of the upper extremities and amputations due to reasons other than limb ischemia, such as venous ulceration, trauma and malignancy, were consistently not included in our present analysis [19,26,27]. Surgical peripheral artery revascularization of the lower extremity comprised peripheral artery bypass operations, incision with embolectomy/thrombectomy and/or patch plastic operations of the legs (OPS codes 5-393.35, 5-393.36, 5-393.38, 5-393.42, 5-393.43, 5-393.44, 5-393.45, 5-393.46, 5-393.5, 5-393.6, 5-393.7, 5-380.7, 5-380.8, 5-395.7, 5-395.8). Peripheral endovascular intervention of the lower extremity comprised all interventional angioplasty treatments including balloon dilatation and stent implantation (OPS codes 8-836.0c, 8-836.0s, 8-836.1c, 8-836.1k, 8-836.2c, 8-836.2k, 8-836.3c, 8-836.3k, 8-840.0c, 8-840.1c, 8-840.2c, 8-840.3c, 8-840.4c, 8-840.5c, 8-840.0s, 8-840.1s, 8-840.2s, 8-840.3s, 8-840.4s, 8-840.5s).

### 2.2. Ethical Aspects

In accordance with German law, approval by the ethical committee and informed consent of the included patients were not required since the study did not involve direct access of the study investigators to the data of individual patients.

### 2.3. Statistical Methods

We compared hospitalization–cases of PAD patients during the treatment year 2019 before the begin of the COVID-19 pandemic in Germany with those hospitalization–cases of PAD patients during the pandemic year 2020. We analysed differences between these groups of two years (before vs. during COVID-19 pandemic) with the help of Wilcoxon–Whitney U test for continuous variables and Fisher’s exact or chi^2^ test for categorical variables, as appropriate.

With this statistically approach, we aimed to analyse the impact and the influence of the COVID-19 pandemic on absolute numbers of admissions of PAD patients, revascularization treatment strategies such as peripheral endovascular intervention of the lower extremity and surgical peripheral artery revascularization of the lower extremity, but also amputations (subclassified as minor and major amputations), in-hospital case fatality, MACCE as well as other adverse in-hospital outcomes in these PAD patients. Additionally, temporal trends in these patients regarding total numbers, mentioned treatments and adverse in-hospital outcomes were investigated. Linear regressions were used to assess trends over time and the results are shown as beta (β) with corresponding 95% confidence intervals (CI).

Statistical significance was presupposed in case of *p*-value < 0.05 (two-sided). Statistical analyses were performed with the software SPSS^®^ (version 20.0; SPSS Inc., Chicago, IL, USA).

## 3. Results

Overall, 361,029 hospitalizations of patients admitted due to PAD in the years 2019 and 2020 (55.4% aged ≥70 years; 36.6% females) were counted and included in the present study. The total number of hospitalizations of patients admitted due to PAD during the pre-pandemic year 2019 was 25,501 (13.2%) admissions higher compared to the COVID-19 pandemic year 2020 (2019: 192,765 [53.4%] vs. 2020: 168,264 [46.6%], *p* = 0.065) (Table 1).

### 3.1. Comparison of Hospitalization Cases of PAD Patients during the Pre-Pandemic Year 2019 versus Hospitalization Cases during the COVID-19 Pandemic Year 2020

While the proportion of admitted patients aged 70 years and older as well as the gender-distribution were similar in both years, patients treated in the COVID-19 pandemic year 2020 showed an aggravated cardiovascular risk profile and a higher proportion of cardiovascular diseases such as coronary artery disease, heart failure and atrial fibrillation/flutter (Table 1). Consecutively, the Charlson comorbidity index revealed an unfavourable patient-profile in patients admitted during the year 2020 in comparison to those hospitalizations during the pre-pandemic year 2019 (5.26 ± 2.23 vs. 5.22 ± 2.23, *p* < 0.001).

Despite the large fear for a hospital-acquired COVID-19 infection, only a small number (610 [0.4%] of the hospitalization–cases) of PAD patients were diagnosed with COVID-19 infection during hospitalization in the year 2020 (Table 1).

Our study demonstrated that the total numbers of both interventional and surgical treatment declined during the COVID-19 pandemic year 2020 in comparison to the pre-pandemic year 2019. While total numbers of peripheral endovascular intervention of the lower extremity decreased substantially from 83,845 in the year 2019 to 75,519 in the year 2020 (*p* < 0.001), the percentage of all annually admitted PAD patients, who were treated with this revascularization strategy, increased from 43.5% in 2019 to 44.9% during the pandemic-year 2020 (Table 1). In addition, the total numbers of surgical peripheral artery revascularization of the lower extremity in hospitalization–cases of PAD patients decreased also significantly from 32,447 in 2019 to 28,754 in 2020 (*p* = 0.041) with similar proportions related to all hospitalization–cases of PAD patients in the mentioned years. Remarkably, the counted numbers of amputations decreased from 2019 to 2020 (20,612 vs. 19,784, *p* < 0.001), whereas the relative numbers increased from 10.7% to 11.8% significantly. Especially the total numbers of minor amputations were affected by the treatment year and the COVID-19 pandemic, while major amputations were widely stable over the mentioned observational period (Table 1).

The case fatality rate (2.6% vs. 2.4%, *p* < 0.001) as well as MACCE rate (3.4% vs. 3.2%, *p* < 0.001) were slightly higher during the pandemic-year 2020 than before the pandemic in the year 2019 (Table 1).

### 3.2. Temporal Trends of Hospitalization, Revascularization Treatments, Amputation Surgeries and Outcomes in PAD Patients over the Observational Period 2019–2020

**Figure 1:**
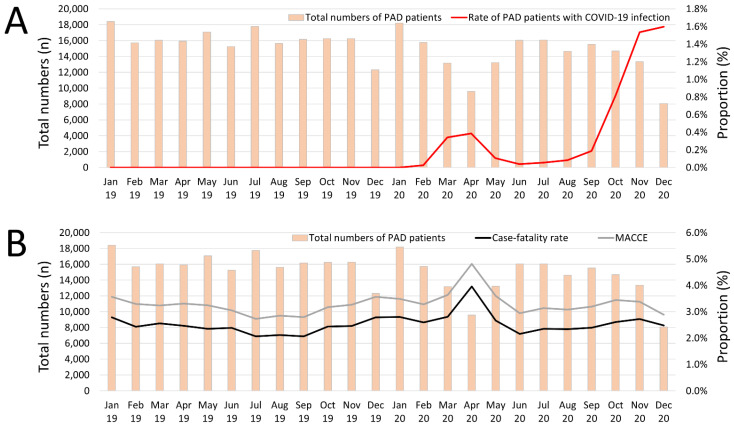
Temporal trends regarding absolute numbers of hospitalizations due to PAD and proportion in these patients with COVID-19 infection, case fatality rate and MACCE rate. (**A**) Temporal trends regarding absolute numbers of hospitalizations of PAD patients (orange bars), and proportion of PAD patients with confirmed COVID-19 infection (orange line) stratified for treatment month. (**B**) Temporal trends regarding absolute numbers of hospitalizations of PAD patients (orange bars), and case fatality rate (black line) as well as MACCE rate (grey line) stratified for treatment month.

Monthly numbers of hospitalizations of PAD patients decreased from 2076 in January 2019 to 1974 in January 2020 and to a minimum in December 2020 with 610 admissions. Nevertheless, this decrease was in the linear regression not statistically significant (β −193.5 [95%CI −437.7 to 50.7], *p* = 0.115) (Figure 1). Illustrated in Figure 1 the relative numbers of hospitalizations of PAD patients with confirmed COVID-19 infection was beyond 0.4% up to September 2020 and increased to 1.6% of all admissions due to PAD in December 2020 (Figure 1A). In contrast, in-hospital case fatality (β 0.130 [95%CI −0.013 to 0.273], *p* = 0.075) and MACCE (β 0.075 [95%CI −0.051 to 0.201], *p* = 0.240) rates were widely stable over the observational period but showed a peak in April 2020 (Figure 1B).

**Figure 2:**
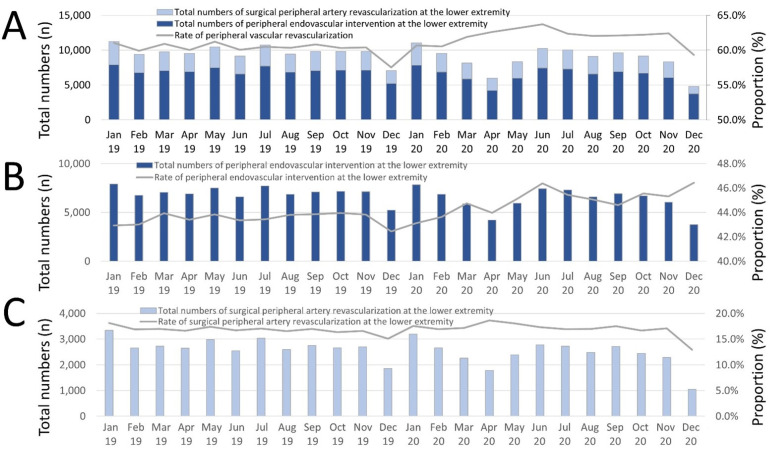
Temporal trends regarding absolute numbers of hospitalizations due to PAD and revascularization treatments. (**A**) Temporal trends regarding absolute numbers of hospitalizations of PAD patients with revascularization treatments (blue bars), and proportion of revascularization treatment related to all hospitalizations of PAD patients (grey line) stratified for treatment month. (**B**) Temporal trends regarding absolute numbers of hospitalizations of PAD patients with peripheral endovascular intervention at the lower extremities (dark blue bars), and proportion of peripheral endovascular intervention at the lower extremities related to all hospitalizations of PAD patients (grey line) stratified for treatment month. (**C**) Temporal trends regarding absolute numbers of hospitalizations of PAD patients with peripheral artery bypass operations (light blue bars), and proportion of peripheral artery bypass operations treatment related to all hospitalizations of PAD patients (grey line) stratified for treatment month.

The temporal trend analyses demonstrated a slight monthly increase regarding peripheral endovascular intervention of the lower extremity (β 0.219 [95%CI 0.174 to 0.264], *p* < 0.0019) (Figure 2B), while the use of surgical peripheral artery revascularization as a treatment approach decreased over time (β −0.065 [95%CI −0.124 to −0.005], *p* = 0.033) (Figure 2C). In contrast, the proportions of peripheral endovascular intervention of the lower extremity increased over time from 2019 to 2020 (Figure 2B), whereas the relative numbers of peripheral artery bypass operations were stable during the observational period (Figure 2C).

**Figure 3:**
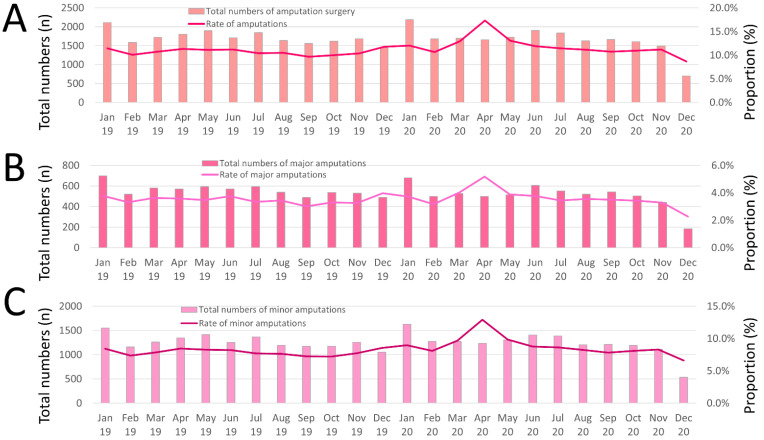
Temporal trends regarding absolute numbers of hospitalizations due to PAD and amputation surgery. (**A**) Temporal trends regarding absolute numbers of hospitalizations of PAD patients with amputation surgeries (red bars), and proportion of amputation treatment related to all hospitalizations of PAD patients (red line) stratified for treatment month. (**B**) Temporal trends regarding absolute numbers of hospitalizations of PAD patients with major amputation surgeries (red bars), and proportion of major amputation treatment related to all hospitalizations of PAD patients (violet line) stratified for treatment month. (**C**) Temporal trends regarding absolute numbers of hospitalizations of PAD patients with minor amputation surgeries (pink bars), and proportion of minor amputation treatment related to all hospitalizations of PAD patients (violet line) stratified for treatment month.

Statistically, monthly numbers of amputations increased over time (β 0.164 [95%CI 0.093 to 0.234], *p* < 0.001) driven by inclining numbers of minor amputations (β 0.189 [95%CI 0.108 to 0.270], *p* < 0.001), but not major amputations (β −0.047 [95%CI −0.168 to 0.074], *p* = 0.447) (Figure 3).

## 4. Discussion

The present study analysing more than 360,000 hospitalizations of PAD patients represents an impact-time-trend investigation regarding the influence of the COVID-19 pandemic on hospitalization numbers, treatment and outcomes of PAD patients. For this objective, we compared the hospital admissions of PAD patients before the beginning of the COVID-19 pandemic in Germany of the year 2019 with those of the COVID-19 pandemic year 2020.

The present key findings can be summarized as follows: I. Overall, 25,501 less hospitalizations of PAD patients were counted in the pandemic year 2020 in comparison to the pre-pandemic year 2019 corresponding to a 13.2% reduction in all hospitalizations of PAD patients. II. Minimal monthly numbers of PAD admissions were detected in winter 2020. III. We observed an unfavourable cardiovascular and comorbidity profile of PAD patients admitted during the pandemic year 2020 in comparison to those in the pre-pandemic year 2019. IV. Only 610 (0.4%) of the patients hospitalized due to PAD were diagnosed with a COVID-19 infection during the year 2020. V. Total numbers of peripheral endovascular intervention as well as surgical peripheral artery revascularization decreased substantially from the pre-pandemic year 2019 to the pandemic year 2020. VI. Although the total numbers of amputations decreased from the pre-pandemic year 2019 to the pandemic year 2020 by 4.0%, the relative annual numbers increased from 10.7% to 11.8%. VIII. Case fatality rate (2.6% vs. 2.4%, *p* < 0.001) as well as MACCE rate (3.4% vs. 3.2%, *p* < 0.001) were slightly higher during the pandemic year 2020 than in the year 2019.

Approximately 200 million people are affected by PAD worldwide [17] with an increasing prevalence [28]. PAD is associated with substantial morbidity and mortality as well as a considerable loss of quality of life [16,19]. Our study results demonstrate a decrease regarding hospital admissions, interventional and surgical revascularization treatments and amputations of PAD patients during the COVID-19 pandemic year 2020. Remarkably, the presented results support the suggestion that PAD patients presented at the hospitals with a significant delay and thus, with a higher severity level of PAD, requiring a higher proportion of interventional and surgical revascularization treatments of the PAD related to the total annual admitted patients and especially a substantially more than 1% higher rate of amputations during the COVID-19 pandemic year 2020.

This suggestion is additionally supported by the aggravated patient-profile, the increased case fatality and MACCE rate during the COVID-19 pandemic year 2020. The in-hospital case fatality excess is obviously not fully explained by the 0.4% hospitalizations of PAD patients suffering from COVID-19.

Our findings are in line with other studies showing that admissions for acute cardiovascular events as well chronic cardiovascular diseases [8,9,10], but also for cancer [11,12,13] as well as mental/behaviour disorders and others [13] were reduced during the COVID-19 pandemic: Although SARS-CoV-2 infection was associated with increased risk for arterial und venous thrombosis [3,29,30,31,32] and patients with MI, who were infected by COVID-19, had an unfavourable outcome in comparison to those MI patients without COVID-19 infection [33], studies observed that admissions for acute MI were significantly reduced during the COVID-19 pandemic [8,9,10] accompanied by increased case fatality rate [8,10,30]. Interestingly, this reduction regarding admissions of MI patients was higher in female patients [8], although studies have reported that especially male sex is a risk factor for adverse outcome in hospitalized COVID-19 patients [3]. In addition, studies have shown that COVID-19 pandemic was associated with an excess of stroke cases in comparison to the pre-pandemic period [31] and the COVID-19 pandemic had remarkable impacts on the management of stroke patients with important delays impeding adequate revascularization therapies [34].

Besides the impact of the COVID-19 pandemic on cardiovascular disease, the cancer diagnosis and treatment [11,12,13] as well as those of mental/behaviour disorders and others [13] are substantially delayed and hampered.

Study results regarding the impact of the COVID-19 pandemic on the hospital admissions of PAD patients and their in-hospital outcomes are very limited [14,35]. In line with our findings, a large epidemiological study investigating health care data of England showed that absolute numbers of amputations and revascularizations of patients with diabetes mellitus were substantially reduced during the pandemic versus the pre-pandemic phase [35]. Other studies revealed that hospitalized PAD patients during the pandemic showed increased severity of PAD and more acute complications [14,15,36]. Although it has been suggested that this reduction regarding the number of admissions during the pandemic in comparison to the pre-pandemic timeframe in patients with cardiovascular diseases and especially PAD patients was probably multifactorial, the most important key factor might be patients’ fear for virus transmission and SARS-CoV-2 contagion as well as COVID-19 infection arising from doctor-patient contacts in the ambulatory setting and/or in particular at the hospitals [8,14]. This fear of hospital-acquired COVID-19 infection is one of the main reasons for patients to avoid hospital admission and thus, PAD diagnostic as well as adequate management is hampered and delayed [14,15]. Another key factor is the change regarding the focus of the health care service [14,15]. In order to encounter the consequences caused by the COVID-19 pandemic, the health care systems have changed the organization and management of the health institutions with focus on the COVID-19 pandemic [4,14,37]. This changed organization had an important impact regarding the diagnosis and treatment of patients with other prevalent, acute or chronic diseases that carry an increased risk for adverse outcomes with and without co-prevalence of COVID-19, but especially in patients with COVID-19 infection, in whom these other acute and chronic diseases might be overlooked, considered to be unimportant at this time or be unnoticed [4,14,37]. These changes in the health care institutions might have contributed to the delays in diagnosis and treatment of PAD patients. This little and limited attention regarding the impact of the COVID-19 pandemic on the admission numbers of PAD patients is contrary to the large burden of PAD worldwide and the influence of PAD on morbidity and mortality [16,17].

The first substantial decrease regarding hospitalizations of PAD patients due to the pandemic was detected in April 2020. During this month, presumably, predominantly PAD patients who could not postpone their hospitalization presented at the German hospitals. In addition, as aforementioned, the German health care system was widely focused on management of COVID-19 patients, resulting in higher case fatality rate, MACCE rate, and higher rate of major and minor amputations of PAD patients than in the months before and after April 2020.

In contrast, despite the surge in COVID-19 cases in December 2020 and the subsequent decrease in hospitalizations for PAD, such a trend with aggravated outcomes was not observed. This finding (in line with the few previous works on the topic) shows that the first wave was really detrimental for these patients, but it also suggests that the management of patients with PAD improved significantly during subsequent waves.

Sena et al. reported that more patients with severe PAD status and especially with chronic limb threatening ischemia presented at their department during the pandemic [14]; for an elevated number of these PAD patients with severe critical limb ischemia with large septic ulcers and gangrene, it was not possible to save the limb and amputation had to be performed [14]. These results are in accordance with our findings showing an increased relative number regarding amputation surgeries during the pandemic in comparison to the pre-pandemic period. Additionally, the increased proportion of the annual number of revascularization treatments related to the annual number of all admitted PAD patients during the COVID-19 pandemic year 2020 point in this direction.

Additionally, the national lockdown measures with stay-at-home orders for longer periods favoured a sedentary life-style with reduced physical activity and exacerbated unhealthy dietary habits resulting in increased prevalence of inactivity, deconditioning and obesity during the pandemic accompanied by psychological side-effects [4,6,37,38,39,40]. The sedentary life-style with its mentioned consequences is a contributing factor for aggravation of claudication and PAD, since treatment strategies in patients with intermittent claudication include exercise training and physical activity as cornerstones of PAD management and prevention of PAD aggravation [41,42]. In this context, healthy diet and physical activity are strongly recommended for all patients with PAD [41].

Regarding PAD and its main driver diabetes mellitus, huge efforts have been made in the past decades to improve therapy approaches including the ongoing development of new medication pathways, the improvement of surgical and interventional procedures as well as the implementation of better patient education and medical surveillance strategies into the regular therapy regime to manage diabetes mellitus and PAD. In disease management programs, the health care providers aimed to manage the patients with improvement of the therapy regime if necessary [19,43,44,45,46]. In our opinion, these management programs have to be expanded regarding COVID-19 specific efforts addressing this critical patient group. In this context, it seems of outstanding interest to encounter in particular the patients’ fear of medical consultations using health management campaigns as well as by the influence of the physicians in charge and in order to identify pandemic-adequate diagnostic and treatment pathways for this vulnerable patient-group to grant rapid access to diagnostic and adequate management including limb-saving revascularization therapies [14,47].

## 5. Conclusions

The COVID-19 pandemic influenced the hospitalization of PAD patients with 13.2% reduction in PAD admissions during the COVID-19 pandemic year 2020 in comparison to the pre-pandemic year 2019. Only 0.4% of the patients hospitalized due to PAD were diagnosed with a COVID-19 infection during hospitalization. Usage of peripheral endovascular intervention of the lower extremity decreased by 9.9%, surgical peripheral artery revascularization of the lower extremity by 11.4% and amputations by 4.0% during the pandemic year 2020 compared to the pre-pandemic year 2019.

## 6. Limitations

Some limitations regarding our study have to be mentioned: Firstly, the present study analysis is based on ICD codes as well we OPS codes of hospitalized patients, which might be prone to under-reporting as well as under-coding. Secondly, detailed baseline data regarding concomitant medications, laboratory markers, and ultrasound parameters are not available in the data-set of the Federal Statistical Office of Germany. Thirdly, due to the structure of the data including only the time-frame of the in-hospital course, follow-up evaluation after discharge was not possible.

## Figures and Tables

**Table 1:** Patients’ characteristics, medical history, presentation and outcome of the included 361,029 hospitalizations of patients admitted due to PAD 2019 and 2020.

Parameters	PAD Patients 2019 (Pre-Pandemic) (*n* = 192,765; 53.4%)	PAD Patients 2020 (during Pandemic) (*n* = 168,264; 46.6%)	*p*-Value
Age (years)	71.90 ± 10.95	72.02 ± 10.90	0.001
Age ≥70 years	106,533 (55.3%)	93,347 (55.5%)	0.204
Female sex	70,570 (36.6%)	61,681 (36.7%)	0.766
Length of in-hospital stay (days)	9.05 ± 11.95	8.66 ± 10.98	<0.001
**Traditional cardiovascular risk factors**			
Diabetes mellitus	66,434 (34.5%)	59,405 (35.3%)	<0.001
Obesity	14,142 (7.3%)	12,190 (7.2%)	0.290
Essential arterial hypertension	131,048 (68.0%)	115,839 (68.8%)	<0.001
Hyperlipidaemia	85,626 (44.4%)	77,720 (46.2%)	<0.001
**COVID-19 Infection**			
COVID-19 infection	-	610 (0.4%)	
Post-COVID-19 status	-	17 (0.01%)	
**Comorbidities**			
Cancer	2954 (1.5%)	2683 (1.6%)	0.133
Coronary artery disease	55,718 (28.9%)	49,287 (29.3%)	0.011
Heart failure	24,611 (12.8%)	21,852 (13.0%)	0.050
Atrial fibrillation/flutter	35,353 (18.3%)	31,706 (18.8%)	<0.001
Chronic obstructive pulmonary disease	19,549 (10.1%)	17,306 (10.3%)	0.155
Acute and chronic kidney disease	57,060 (29.6%)	50,130 (29.8%)	0.209
Chronic renal insufficiency (glomerular filtration rate < 60 mL/min/1.73 m^2^)	42,023 (21.8%)	36,937 (22.0%)	0.271
Charlson comorbidity index	5.22 ± 2.23	5.26 ± 2.23	<0.001
**Revascularization and Amputation treatment**			
Amputation	20,612 (10.7%)	19,784 (11.8%)	<0.001
Minor amputation	15,176 (7.9%)	14,743 (8.8%)	<0.001
Major amputation	6708 (3.5%)	6057 (3.6%)	0.052
Peripheral endovascular intervention of the lower extremity	83,845 (43.5%)	75,519 (44.9%)	<0.001
Surgical peripheral artery revascularization of the lower extremity	32,447 (16.8%)	28,754 (17.1%)	0.041
**Adverse events during hospitalization**			
In-hospital death	4631 (2.4%)	4401 (2.6%)	<0.001
MACCE	6105 (3.2%)	5678 (3.4%)	<0.001
Cardio-pulmonary resuscitation	1211 (0.6%)	1159 (0.7%)	0.025
Shock	1833 (1.0%)	1716 (1.0%)	0.036
Myocardial infarction	1346 (0.7%)	1246 (0.7%)	0.134
Pulmonary embolism	242 (0.1%)	195 (0.1%)	0.405
Deep venous thrombosis or thrombophlebitis	1076 (0.6%)	948 (0.6%)	0.834
Pneumonia	3914 (2.0%)	3601 (2.1%)	0.021
Acute kidney injury	6767 (3.5%)	6432 (3.8%)	<0.001
Stroke (ischaemic or haemorrhagic)	720 (0.4%)	613 (0.4%)	0.649
Intracerebral bleeding	62 (0.03%)	58 (0.03%)	0.705
Gastro-intestinal bleeding	938 (0.5%)	899 (0.5%)	0.045
Transfusion of blood constituents	17,625 (9.1%)	16,441 (9.8%)	<0.001

## Data Availability

Statistical analyses were performed on our behalf by the Research Data Center (RDC) of the Federal Bureau of Statistics (Wiesbaden, Germany) (source: RDC of the Federal Statistical Office and the Statistical Offices of the federal states, DRG Statistics 2019–2020, and own calculations).

## Abbreviations

CI	Confidence interval
COVID-19	Coronavirus disease 2019
DRG	Diagnosis Related Groups
ICD	International Classification of Diseases and Related Health Problems
OPS	Diagnostic, surgery and procedures codes (Operationen- und Prozedurenschlüssel)
MACCE	Major adverse cardiovascular and cerebrovascular events
MI	Myocardial infarction
PAD	Peripheral artery disease
RDC	Research Data Center
